# Adsorption Properties of Modified Clinoptilolite for Methane and Nitrogen

**DOI:** 10.3390/ma11102024

**Published:** 2018-10-18

**Authors:** Xiaofei Hao, Hongjie Hu, Zhen Li, Limei Wu, Xueqin Liu, Yinnian Zhang

**Affiliations:** 1 Engineering Research Center of Nano-Geomaterials of Ministry of Education, Faculty of Materials Science and Chemistry, China University of Geosciences, Wuhan 430074, China; goodpaper@cug.edu.cn (X.H.); liuxq@cug.edu.cn (X.L.); 2 Zhengzhou Fulong Science and Technology of New materials Co., Ltd., Zhengzhou Institute of Multipurpose Utilization of Mineral Resourse, China Academy of Geological Sciences, Zhengzhou 450006, China; 13608685651@139.com (H.H.); yinnianz1234@sina.com (Y.Z.); 3 School of Materials Science and Engineering, Shenyang Jianzhu University, Shenyang 110168, China; lmwu@sjzu.edu.cn

**Keywords:** clinoptilolite, ion-exchange, nitrogen/methane separation.

## Abstract

Coalbed methane (CBM) is a kind of unconventional gas. CBM often contains a great deal of air when it comes out of the well. So, it must be condensed and purified before it can be applied. In this paper, raw clinoptilolite (Cp) was treated with grinding, gravimetric concentration, and ion-exchange using different aqueous solutions of salts. Then, the modified Cp powder was prepared into particles as adsorbents. Then, the adsorbents were used for nitrogen/methane separation in pressure swing adsorption (PSA) at the same condition of 0.2 MPa and 298 K. Research results indicated that there were micropores and lots of mesopores in the Cp, and the pores were mainly slit holes formed by sheet stacking. The adsorbents of NH_4_-Cp, Cs-Cp, and Cu-Cp showed good equilibrium selectivity for CH_4_, and the equilibrium separation factors of CH_4_ and N_2_ were 2.56, 2.31, and 1.95, respectively. The adsorbents of Na-Cp and Ag-Cp showed good equilibrium selectivity for N_2_, and the equilibrium separation factors of N_2_ and CH_4_ were 7.25 and 6.53, respectively. Consequently, the adsorbent of Na-Cp was suitable for nitrogen/methane mixture separation, which could make the concentration of methane concentrated from 19.7% to 30.72%.

## 1. Introduction

For a long time, coalbed methane (CBM) has been discharged into the atmosphere from coal mines for the safe of coal production. In recent years, in the face of a lack of energy and the pressure of environmental pollution, especially in China, more and more people have gradually realized that CBM should be tapped as an unconventional natural gas and realize the control of atmospheric pollutants at the same time. CBM, which contains significant amounts of nitrogen (N_2_ > 70%, CH_4_ > 20%), needs to be upgraded in order to meet the quality for civil use (CH_4_ ≥ 30%). The mature technology is cryogenic distillation for nitrogen removal from methane, but it is highly energy-intensive and costly. An alternative way of separating the CH_4_/N_2_ mixture is pressure swing adsorption (PSA) [1,2,3,4]. The selection of appropriate adsorbent is crucial to the successful separation of methane and nitrogen using PSA.

Up to now, the main adsorbents are activated charcoal, carbon molecular sieve (CMS), and zeolite molecular sieve. The equilibrium adsorption capacity of activated carbon to methane is higher than that of nitrogen, and the separation coefficient is higher. However, in the application process, activated carbon is easily powdered, which often causes blockage of equipment. So, it characterized by large gas circulation, low efficiency, and high costs. The CMS is mainly based on kinetic effect for separation of nitrogen and methane. That is to say, the diffusion rate of nitrogen is higher than that of methane in the micropore of CMS, and a large amount of nitrogen is adsorbed into the pore in a short time, and methane remains outside the pore. The concentrated methane is obtained in the sequence step. However, with the increase of adsorption time, the kinetic effect becomes weaker and the equilibrium effect plays a key role. Then, the equilibrium effect coexists with the kinetic effect, and their separation effect is the opposite. Accordingly, there is difficulty in separating methane and nitrogen. The zeolite molecular sieve is a very promising adsorbent. Zeolites, because of their inherent porosity, crystallinity, and the presence of hydrated aluminosilicates of alkali and alkaline earth cations, can absorb polar molecules and small molecules: they have long been considered as excellent candidate materials for the separation of gases. According to their classification, zeolites occur as one of two types: synthetic zeolites and natural zeolites. Synthetic zeolites are manufactured on a large scale for industrial use. However, compared with synthetic zeolites, natural zeolites are abundant and readily available. Moreover, they are cheap. Therefore, they have been paid attention increasingly by people.

**Figure 1:**
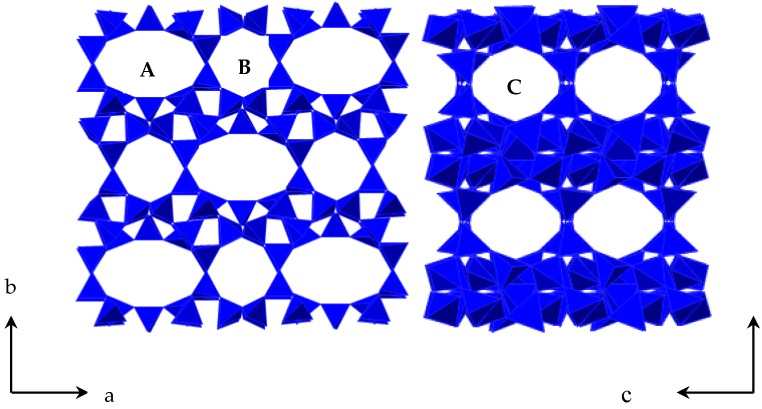
The structure of Cp.

Clinoptilolite (Cp) is a member of heulandite group of natural zeolites and is isostructural with the zeolilte heaulandite. The general formula for natural zeolites is (Na, K)*_a_*(Mg, Ca, Sr, Ba)*_d_*[Al*_a_*_+2*d*_Si*_n_*_−(*a*+2*d*)_O_2*n*_]·*m*H_2_O. The unit cell is monoclinic and is usually characterized on the basis of 72 O atoms (*n* = 36) and *m* = 24 water molecules. The framework of Cp is formed by two parallel channels of 10-membered rings (channel A, 0.72 × 0.44 nm) and 8-membered rings (channel B, 0.47 × 0.41 nm) that are connected to the other 8-membered rings (channel C, 0.55 × 0.40 nm) [5]. A schematic diagram of Cp structure is given in Figure 1. In these channels, there was both zeolite water and metal cations: these (K^+^, Na^+^, Ca^2+^, and Mg^2+^) balance the negative charge in the lattice and are readily exchanged with cations in aqueous solution (such as K^+^, Sr^2+^, Ba^2+^, etc.). They do not destroy the crystal structure but can change the electric field within the crystal; thus, the adsorption properties of the Cp can be changed to a significant extent. Cp has been studied with a view to its use in natural gas purification. Aguilar-Armenta et al. measured the adsorption kinetics of pure CO_2_, N_2_, and CH_4_ on a natural clinoptilolite sample from Villa de Reyes [6]. Jayaraman et al. found that purified and Mg-clinoptilolite showed potential for nitrogen/methane separation [7]. Faghihian et al. investigated the adsorption of N_2_ and CH_4_ on natural Cp from the north of Semnan and its cation-exchanged forms (Na-Cp, K-Cp, and H-Cp) at 298 K [8]. However, it is hard to enrich methane through kinetic PSA, since the methane content has been lower than 20% in the feed gas until now.

In this paper, in view of the fact that large deposits of zeolitic tuffs, primarily of Cp, have been discovered in China, the adsorption properties of modified Cp (using aqueous solutions of salts KCl, NaCl, AgNO_3_, RbCl, CsCl, LiCl, NH_4_Cl, CaCl_2_, MgCl_2_, BaCl_2_, SrCl_2_, CuCl_2_, ZnCl_2_, and CeCl_3_) was investigated to evaluate their possible industrial applications in CBM separation processes. The simulated methane–nitrogen mixture (19.7% CH_4_, 80.3% N_2_) was separated using a single-bed unit with the modified Cp in the same conditions of adsorption pressure and temperature in the PSA equipment.

## 2. Materials and Methods

### 2.1. Materials

The natural clinoptilolite (Cp) was collected from the south of the Liaoxi metallogenic belt in China, and its structure was shown in Figure 1. Its chemical composition (wt.%) is SiO_2_ 68.48%, TiO_2_ 0.19%, Fe_2_O_3_ 1.35%, Al_2_O_3_ 11.92%, CaO 3.75%, MgO 1.26%, K_2_O 1.61%, and Na_2_O 0.63%. The natural clinoptilolite was crushed to 200 mesh by wet-grinding, and the milled pulp was poured into a centrifuge to remove any heavy impurities. Then, purified clinoptilolite (p-Cp) was obtained with the following chemical composition (wt.%): SiO_2_ 66.99%, TiO_2_ 0.20%, Fe_2_O_3_ 1.37%, Al_2_O_3_ 12.01%, CaO 3.80%, MgO 1.29%, K_2_O 1.63%, and Na_2_O 0.65%. This process differed from that quoted in the existing literature [6,7,8] in that the purified clinoptilolite was dried at 378 K and stored in a desiccator. It was used as a raw material for the preparation of adsorbents.

Helium (99.999%, pre-purified, Praxair Gases), nitrogen, and methane were used as adsorbates. Helium was used as a purge gas during desorption. All gases were supplied by Praxair (Beijing, China). Other used analytical-grade chemicals were purchased from Sinopharm Chemical Reagent Co., Ltd. (Shanghai, China). Deionized water was used for sample washing, and silver nitrate was used to check for the absence of Cl^−^ ions in the washings.

### 2.2. Preparation of Modified Clinoptilolites

The samples were prepared from p-Cp by ion exchange with 2 M aqueous solutions of respective salt KCl, NaCl, AgNO_3_, RbCl, CsCl, LiCl, NH_4_Cl, CaCl_2_, MgCl_2_, BaCl_2_, SrCl_2_, CuCl_2_, ZnCl_2_, and CeCl_3_ in a three-neck flask in a 90 °C water bath for 2 h on a magnetic stirrer. The above-mentioned all salt solutions were mixed by the weight of the solid to liquid ratio at 1:20. This procedure was repeated three times until the cations contents were no longer changed in the filtrate. After these, the mixture was filtered and washed many times with deionized water until white precipitates disappeared in filtrate by test with silver nitrate. Finally, the samples were through simple processing, drying, and fine grinding, and they were put in a glass dryer for use. All samples were depicted by K-Cp, Na-Cp, Ca-Cp, Mg-Cp, Li-Cp, Rb-Cp, Sr-Cp, Ba-Cp, Ce-Cp, Cu-Cp, Zn-Cp, Cs-Cp, Ag-Cp, H-Cp which were treated by KCl, NaCl, CaCl_2_, MgCl_2_, LiCl, RbCl, SrCl_2_, BaCl_2_, CeCl_3_, CuCl_2_, ZnCl_2_, CsCl, AgNO_3_, and NH_4_Cl solution, respectively. Then, the modified Cppowder was prepared into particles as adsorbents between 0.5 and 1.5 mm.

### 2.3. Materials Characterization

To detect possible structural changes produced by thermal activation, X-ray diffraction patterns of the natural, purified, and cation-exchanged Cp were assessed. X-ray powder diffraction (XRD) patterns of the clinoptilolite samples were obtained from a MiniFlex 600 (Rigaku, Japan) diffractometer using CuK_α_ radiation (*λ* = 1.5406 Å). The FTIR spectrum of each sample was obtained by using a Nicolet (IS50) spectrophotometer (Thermofisher, Waltham, MA, USA). The silicon content of all samples was analyzed by ultraviolet visible spectrophotometer (SPECORD 200, Analytik Jena, Jena, Germany). The rubidium and silver content of all samples was analyzed by atomic absorption spectrophotometry (SOLAAR M6, Thermofisher, Waltham, MA, USA). Other analysis of chemical components of all samples was analyzed by inductively coupled plasma atomic emission spectrometry (iCAP 7400, Thermofisher, Waltham, MA, USA). The surface morphology and size of sample was observed by SEM (SU8020, Hitachi, Tokyo, Japan). The polarizing microscope (ZEISS Axio Scope A1, Oberkochen, Germany) with plane-polarized light revealed the phase composition.

Static adsorption isotherms of samples at low temperatures with argon gas at 87.27 K (Autosorb iQ, Quantachrome, Boynton Beach, FL, USA) were obtained: the static adsorption properties of adsorbents for methane and nitrogen were studied with a static volumetric system (BELSORP-miniII, BEL, Japan). Before measurement, the Cp samples were activated at 573 K in an oven evacuated to a residual pressure of less than 3.2 × 10^−2^ Pa and maintained in those conditions for 6 h. The static adsorption characteristics of Cp for nitrogen or methane were then tested at 298 K.

Molecular simulation was performed under the module ‘CASTEP’ of Materials Studio 7.0 software (Accelrys, San Diego, CA, USA) to investigate the sorption sites of cation on Cp. The primitive unit cell of Cp was optimized with the generalized gradient approximation (GGA) for the exchange-correlation potential (PW91). The resulting primitive unit cell was characterized by the parameters a = 17.77 Å, b = 17.95 Å, c = 7.44 Å, and α = γ = 90°, and β = 116°. The number of cycles is 3, and the steps of one cycle are 10^6^, a representative part of the interface devoid of any arbitrary boundary effects.

### 2.4. Dynamic Separation Experiment with CH_4_/N_2_

**Figure 2:**
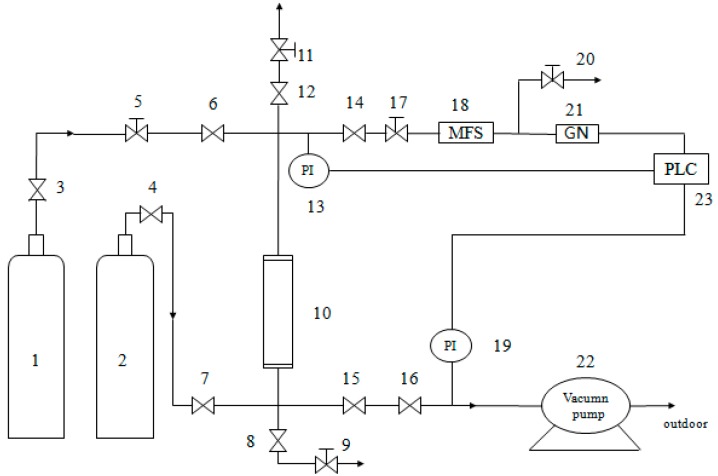
The dynamic test device. 1: He cylinder; 2: Mixture cylinder of CH_4_/N_2_; 3 and 4: Pressure reducing valve; 5, 9, 11, 17 and 20: Needle-type valve; 6, 7, 8, 12, 14, 15 and 16: Common valve; 10: Absorbing tower (16 mm × 320 mm); 13 and 19: Pressure probe; 18: Mass flow controller; 21: Gas analyzer; 22: Vacuum pump; 23: Windows control center (Copyright^©^1994–1997 SIEMENS, Germany).

The feed gas is the mixture gas of CH_4_ and N_2_, and the volume ratio of CH_4_/N_2_ was 19.7%: 80.3%. The simulated methane-nitrogen mixture (19.7% CH_4_, 80.3% N_2_) was separated using a single-bed unit (Shown as Figure 2) with the modified Cp in the same conditions of 0.2 MPa and 298 K in the PSA equipment (self-assembly). The absorbing tower was filled with p-Cp and modified Cp, respectively. At first, the device was pressurized using high-pure standard He until the adsorption pressure was up to the setting pressure at 0.2 MPa. Then, the intake valve of He was closed, and the intake valve of mixture gas was opened (it was the start time of data recording). In order to keep the pressure of absorbing tower reaching the experimental value, it was adjusted by control valves (the flow value of gas was set to 60 mL/min). The outlet discharge was set using mass flow controller before the test. In the process of absorption, the change of concentration of CH_4_ was tested and recorded by gas analyzer. The testing was not over until the testing volume concentration of CH_4_ had risen to the concentration of CH_4_ in the feed gas. Then, all valves were closed, and testing was over. Finally, the activation and regeneration of modified clinoptilolite was not begun until inverse vacuum was pumped for 30 min.

## 3. Result and Discussions

### 3.1. Characterization of Cp and Modified Clinoptilolites

**Figure 3:**
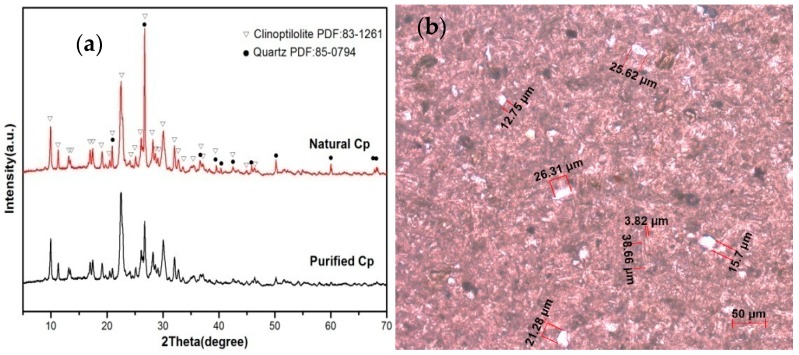
XRD patterns of natural Cp and purified Cp (**a**); the polarizing microscope with plane-polarized light showing the phase composition (**b**).

The XRD patterns of the natural Cp sample showed the main crystalline phases were quartz and Cp (Figure 3a). The polarizing microscope with plane-polarized light showed the crystals as either a Cp phase or a quartz phase (white granular crystals, whose size is 15.7 to 29.02 μm) (Figure 3b). The natural Cp was purified, and the Cp-content exceeded 90 wt.%, so approximately 10% of the original material had been removed as impurities. From the analysis of the chemical composition, a reduction of silica quartz was also seen, and this was consistent with the chemical composition analysis. The XRD pattern of purified Cp showed that the characteristic peaks of quartz at 2θ values (in degrees) of 20.92, 26.70, 39.54, 40.36, 42.50, 50.18, 54.92, 60.00, 67.76, and 68.34 had vanished, so the sample was deemed to have been purified (Figure 3a).

**Figure 4:**
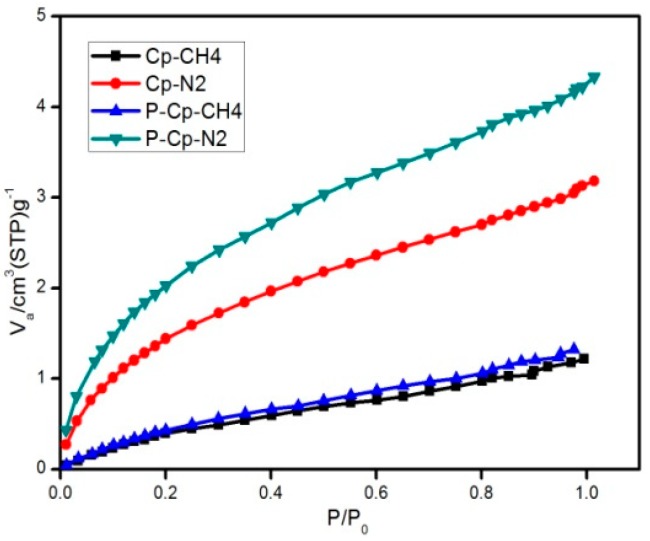
Low-pressure nitrogen and methane isotherms on raw and purified Cp at 298 K.

The N_2_ and CH_4_ adsorption isotherms of Cp and purified Cp are shown in Figure 4. After purification, the adsorbed CH_4_ amount hardly increased at all. In contrary, the adsorbed N_2_ amount increased significantly. This will greatly improve the selectivity adsorption of N_2_ and increase the CH_4_ concentration of products.

**Figure 5:**
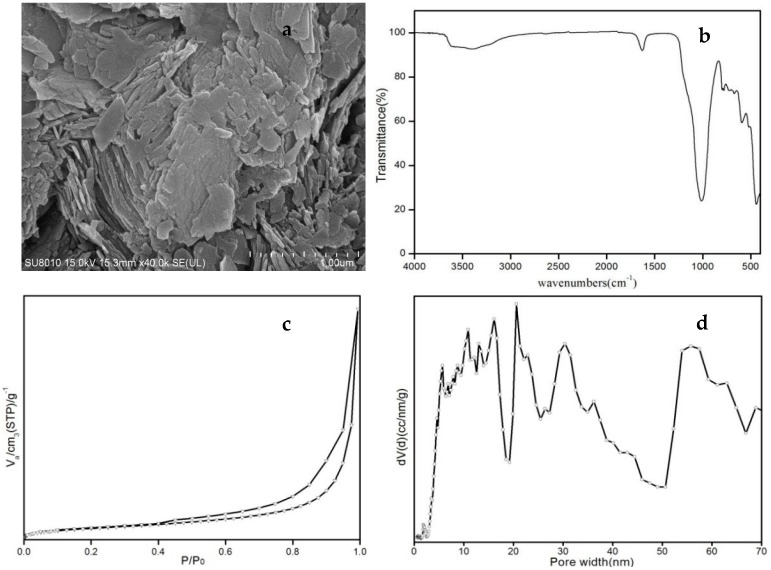
SEM photomicrograph of the glassy surface of p-Cp (**a**), FTIR spectrum of p-Cp (**b**), adsorption isotherms of p-Cp (**c**), and pore-size distribution of p-Cp (**d**).

A scanning electron micrograph (SEM) of the classic crystalline morphology of Cp showed the morphology of a glassy, flaky surface is shown (Figure 5a). The FTIR spectrum of the purified Cp was shown (Figure 5b). The peaks located at 3366 and1628 cm^−1^ were related to stretching modes of O-H, the peaks at 1011 cm^−1^ arise as a result of asymmetrical stretching, and the peaks at 440, 591, 670, and 780 cm^−1^ arise as a result of the symmetrical stretching of SiO_4_ and AlO_4_, respectively. According to IUPAC, the static adsorption isotherms of Cp correspond to blend types IV (Figure 5c). In the low-pressure *P*/*P*_0_ region, monolayer adsorption occurred, and in the higher-pressure *P*/*P*_0_ region, multi-layer adsorption occurred. The adsorption isotherms increased rapidly with respect to pressure ratio *P*/*P*_0_. The samples showed evidence of slit hole structures due to the aggregation of flaky particles. The hysteresis loop corresponds to type H_3_. There were micropores, mesopores, and macropores in the samples, and the pore-size distribution is shown (Figure 5d). Its multipoint BET was 18.92 m^2^/g, and DFT method cumulative pore volume was 0.059 cc/g. The proportion of micropores (1–2 nm) was 0.15%, and mesopores (4–38 nm) and macropores (55–65 nm) were 0.15%, 88.88%, and 10.97%, respectively.

### 3.2. Characterization of Adsorbents 

Table 1 shows the composition (wt.%) of the purified and modified Cp, and the exchange capacity of each cation Cp. The degree of exchange for a cation was calculated using *D*_i_ = 100*C*_i_/*C*_0_, in which *C*_i_ is the number of equivalents of i extracted, and *C*_0_ is the number of equivalents of i present in the original sample. The contents of Fe_2_O_3_ and TiO_2_ basically remain the same in different types of cationic Cp. From the data in Table 1, the ability to exchange calcium ions in Cp decreased in the order of NH_4_-Cp > Rb-Cp > K-Cp > Na-Cp > Ag-Cp > Cs-Cp > Li-Cp > Cu-Cp > Zn-Cp > Ba-Cp > Sr-Cp > Mg-Cp > Ce-Cp. The ability to exchange sodium ions in C_p_ decreased in the order of Rb-Cp > K-Cp > Cs-Cp > Sr-Cp ≈ Ag-Cp > Ba-Cp > Ca-Cp > NH_4_-Cp > Mg-Cp > Cu-Cp > Ce-Cp > Li-Cp > Zn-Cp. The ability to exchange potassium ions in Cp decreased in the order of Rb-Cp > NH_4_-Cp > Cs-Cp > Ag-Cp > Ba-Cp > Na-Cp > Sr-Cp > Ca-Cp ≈ Cu-Cp > Li-Cp > Zn-Cp > Mg-Cp > Ce-Cp. The ability to exchange magnesium ions in CP decreased in the order of NH_4_-Cp > K-Cp > Rb-Cp ≈ Ag-Cp > Cs-Cp > Na-Cp > Li-Cp > Cu-Cp > Zn-Cp > Ba-Cp > Ca-Cp > Sr-CP > Ce-CP. The ability to absorb water of crystallization in C_p_ decreased in the order of NH_4_-Cp > Mg-Cp > Cu-Cp > Ce-Cp > Ca-Cp > Sr-Cp > Li-Cp > Zn-Cp > Ba-Cp > Na-Cp > Ag-Cp > K-Cp > Rb-Cp > Cs-Cp. Due to the polar properties of H_2_O, the adsorption of water was correlated with the ions polarization forces. Moreover, because of the amounts of H^+^ in the Cp, Mg^2+^, Sr^2+^, Ba^2+^, Ce^3+^, Cu^2+^, and Zn^2+^ exchanges were blocked, which exhibited a weak exchange ability.

**Figure 6:**
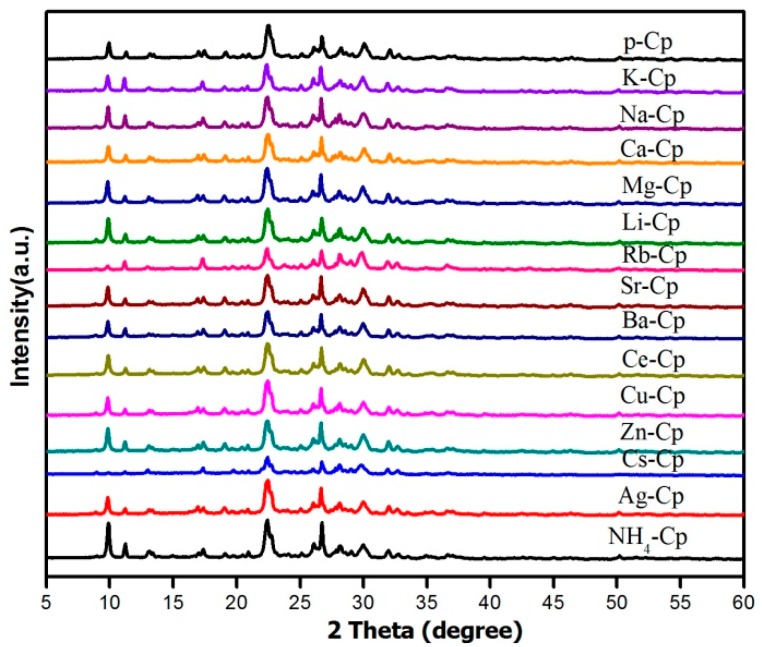
The XRD patterns of the modified Cp.

The XRD patterns of the modified Cp were shown in Figure 6. It is obvious that the main crystalline phases corresponded to Cp, and their crystal structures were unchanged after modification. However, a closer inspection revealed that the (020) faces of Rb^+^ and Ce^3+^-modified Cp were very weak. It was deduced that these ions may have been able to replace aluminum in the structure, which caused the decreased degree of crystallization.

The size of the adsorbate molecule must be smaller than the channel dimension of the Cp. The diameters for N_2_ and CH_4_ are 0.364 nm and 0.38 nm, so it is easier for nitrogen to enter the channel than methane. When the channels are open enough to allow the free circulation of the adsorbate, the adsorptive capability is related to the electrostatic interaction of the molecule with the adsorption centers of the zeolite. The strength of such an interaction depends mainly on the following two factors [9]: the overall intensity of the local electrostatic field originating from the ionic nature of the zeolite framework, and the polarity (dipole and quadrupole moments) and/or polarizability (induced dipole) of the adsorbate. In addition, depending on the structure and topology of the zeolite, the volume and location of the counter-cations may significantly affect the adsorption capabilities. When the adsorption sites are not hindered, the lower the energy of the system, the more stable it would be. Therefore, the adsorption capability of CH_4_ is greater than that for N_2_.

**Figure 7:**
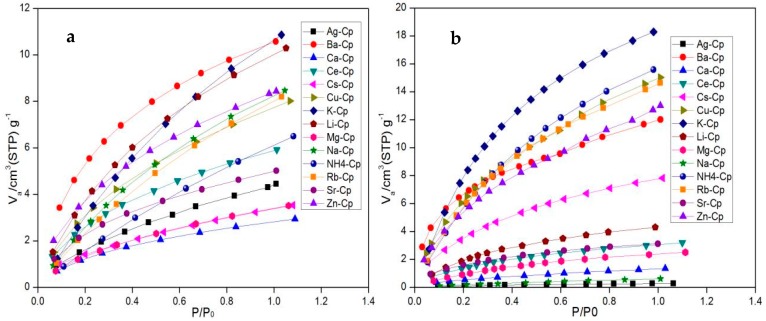
Low-pressure nitrogen (**a**) and methane (**b**) isotherms on modified Cp at 298 K.

The N_2_ and CH_4_ adsorption isotherms of modified samples are shown in Figure 7; the CH_4_ adsorption uptakes decrease in the order: K-Cp, NH_4_-Cp, Rb-Cp, Cu-Cp, Zn-Cp, Ba-Cp, and Cs-Cp, and they are methane-selective. Despite the non-polar nature of methane, this molecule interacts strongly with the zeolite due to its high polarizability, in such a way that its interaction energy is higher than the interaction energy for N_2_. With regard to an N_2_ molecule, the N_2_ adsorption uptakes decrease in the order: Li-Cp, Na-Cp, Ce-Cp, Sr-Cp, Ag-Cp, Mg-Cp, and Ca-Cp, and they are nitrogen-selective due to the inherent quadrupole in the N_2_, and the pore size. The adsorption capacity of CH_4_ and N_2_ of all samples was shown in Table 2. The adsorbents of NH_4_-Cp, Cu-Cp, and K-Cp show good equilibrium selectivity for CH_4_, and the equilibrium separation factors of CH_4_ and N_2_ were 2.56, 1.95, and 1.82, respectively. The adsorbents of Na-Cp and Ag-Cp show good equilibrium selectivity for N_2_. The equilibrium separation factors of N_2_/CH_4_ were 7.25 and 6.53, respectively. In addition to the heat of adsorption, the pore volume is also an important factor affecting the amount of adsorption.

**Figure 8:**
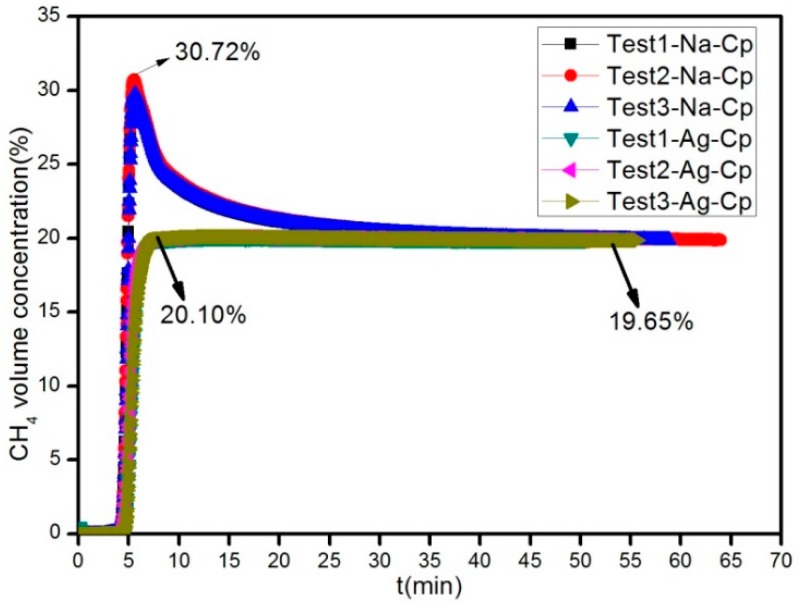
The Na-Cp and Ag-Cp breakthrough curve of nitrogen adsorption.

Dynamic experiments had been done. The CH_4_ volume concentration of product is obtained at 298 K on 0.2 MPa when the feed gas is a mixture of CH_4_ (19.7%) and N_2_ gas (80.3%), as shown in Figure 8. Concentrated CH_4_ could be obtained directly by using the adsorbents of Na-Cp, Ag-Cp. In the dynamic adsorption curve of Na-Cp, it showed that CH_4_ concentration can be increased from 19.7% to 30.72%. Moreover, it can be continuously regenerated. Ag-Cp showed that CH_4_ concentration can be increased from 19.7% to 20.10%. The performance of adsorbent Ag-Cp is not obvious, mainly due to the lower adsorption capacity, which is consistent with the static adsorption results.

### 3.3. Adsorption Mechanism

In order to get a better understanding of above phenomena, computer molecular simulation was used to investigate the silicon tetrahedron micro structure and the cation interaction for this Cp at atomic scale. Based on force-field, equilibrium molecular dynamics simulations were performed. The interactions between structure silicon tetrahedron and cations were calculated by combining the parameters of noncovalent energy terms. The combined force field has been proven to be able to maintain the structures of both clay mineral and cations, and guarantees full interactions among them [10,11,12]. The simulated system had its total energy as the combination of Coulombic interaction, van der Waals interaction, and bonded interaction:

$$\;E_{total}=E_{VDW}+E_{Coulombic}+E_{bond\_stretch}+E_{angle\_bend}+E_{torsion}+E_{improper}\;$$

The first two terms of the total energy contributed to the Lennard–Jones potential (12–6 potential) and Coulombic potential energy terms, and the sum of them, represented the noncovalent interaction that is universal for any two atoms. For bonded interactions, the energy terms that compensate bond stretch, angle bend, torsional, and improper movements were considered. All the bonded terms were calculated based on CLAYFF.

**Figure 9:**
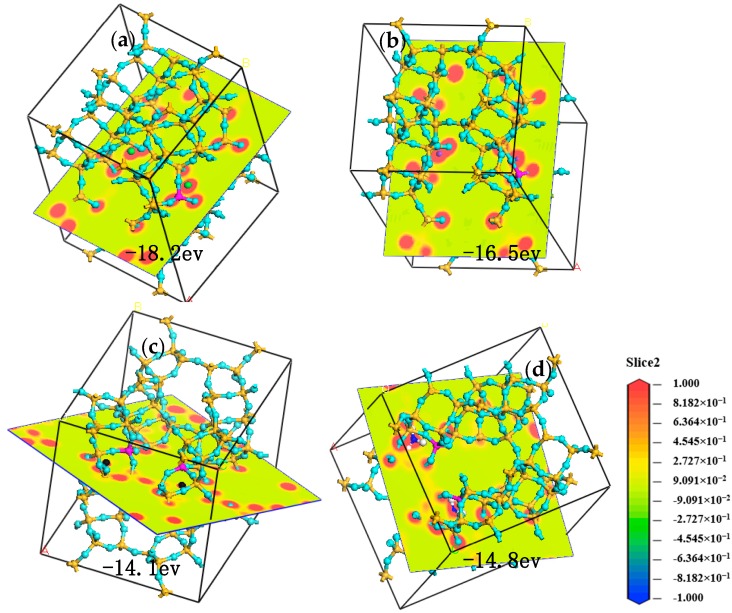
Molecular dynamic simulation of K-Cp (**a**), Li-Cp (**b**), Ca-Cp (**c**), and NH_4_-Cp (**d**). For all species, O = light blue, H = white, Si = yellow, Al = deep powder, K = green, Li = purple, and Ca = black and N = blue.

Cation plays an important role in Cp function, as variation of cation species leads to different electric field, thus rendering great differences in adsorption performance. Meanwhile, some cations always block channel of Cp crystals and change effective aperture. Different adsorption performance to CH_4_ and N_2_ of Cp with different cations is mostly due to interaction force between cations and silicon-aluminum structure and chemical bond [13]. Higher structural energy means stronger molecular interaction force, which decreases adsorption rate and capacity and finally leads to adsorptive selectivity. The cations would exist in the interlayer in the form of Xn^+^-mH_2_O. Methane and nitrogen are nonpolar molecules. Nitrogen has a quadrupole, while methane does not. As shown in Figure 9, the binding energies for Li^+^, K^+^, Ca^2+^, and NH_4_^+^ with aluminum oxide tetrahedron are −16.5, −18.2, −14.1, and −14.8 eV, respectively. The force between K^+^ and aluminum oxide tetrahedron is the strongest, so the highest of the adsorption for N_2_. Ca^2+^ has the weakest force with aluminum oxide tetrahedron, and thus the lowest of the adsorption for N_2_. The amount of bound water around cations and the interaction force between them affected the adsorption ability of the Cp. During the cation exchange, the cations go into the interspaces between the crystal skeleton of O ions formed in Cp. It would inevitably involve the diffusing out of cations and diffusing in of the CH_4_ or N_2_.

## 4. Conclusions

In conclusion, natural clinoptilolite with micropores, mesopores, and macropores on its surface was purified using centrifugal concentrator. The clinoptilolite adsorbents prepared via the ion exchange treatment of purified clinoptilolite with K^+^, NH_4_^+^, Rb^+^, Cu^2+^, Zn^2+^, Ba^2+^, Cs^+^, Li^+^, N^+^, Ce^3+^, Sr^2+^, Ag^+^, Mg^2+^, and Ca^2+^ showed different adsorption properties for CH_4_ and N_2_. The adsorbents of NH_4_-Cp, Cu-Cp, and K-Cp showed strong adsorbability for CH_4_, and the equilibrium separation factors for CH_4_/N_2_ were 2.56, 1.95, and 1.82, respectively. Moreover, the adsorbents Cp-Na and Cp-Ag showed good absorbability for N_2_, and the equilibrium separation factors for N_2_/CH_4_ were 7.25 and 6.53, respectively. Lastly, through the dynamic simulation test of CH_4_ and N_2_, it was found that best sorbent is Na-Cp, which produced high concentration CH_4_. The large equilibrium separation factor and smaller investment make the practical application of adsorbent Cp-Na possible. 

## Figures and Tables

**Table 1:** Composition (wt.%) of the purified and modified Cp.

Sample	Composition (%)														Exchange (%)			
	K_2_O	Na_2_O	CaO	MgO	Rb_2_O	Ce_2_O_3_	Li_2_O	BaO	SrO	CuO	Ag_2_O	ZnO	Cs_2_O	H_2_O	K	Na	Ca	Mg
P-Cp	1.63	0.65	3.80	1.29	bd	bd	bd	bd	bd	bd	bd	bd	bd	9.29	/	/	/	/
K-Cp	9.97	0.31	0.29	0.51	bd	bd	bd	bd	bd	bd	bd	bd	bd	6.06	/	52.3	92.4	60.5
Na-Cp	1.41	5.43	0.48	0.69	bd	bd	bd	bd	bd	bd	bd	bd	bd	8.21	13.5	/	87.4	46.5
Ca-Cp	1.50	0.39	4.32	1.20	bd	bd	bd	bd	bd	bd	bd	bd	bd	9.53	8.0	40.0	/	7.0
Mg-Cp	1.62	0.48	3.38	1.84	bd	bd	bd	bd	bd	bd	bd	bd	bd	9.87	0.6	26.2	11.1	/
Li-Cp	1.51	0.37	1.71	0.82	bd	bd	1.53	bd	bd	bd	bd	bd	bd	9.20	7.4	4.3	55.0	36.4
Rb-Cp	1.01	0.29	0.27	0.53	14.46	bd	bd	bd	bd	bd	bd	bd	bd	5.21	38.0	55.4	92.9	58.9
Sr-Cp	1.48	0.35	3.10	1.23	bd	bd	bd	bd	2.74	bd	bd	bd	bd	9.48	9.2	46.2	18.4	4.7
Ba-Cp	1.39	0.36	3.00	1.18	bd	bd	bd	3.76	bd	bd	bd	bd	bd	9.08	14.7	44.6	21.1	8.5
Ce-Cp	1.65	0.59	3.59	1.30	bd	0.85	bd	bd	bd	bd	bd	bd	bd	9.70	/	9.2	5.5	/
Cu-Cp	1.50	0.52	2.20	0.88	bd	bd	bd	bd	bd	3.35	bd	bd	bd	9.85	8.0	20.0	42.1	31.8
Zn-Cp	1.71	0.64	2.56	1.04	bd	bd	bd	bd	bd	bd	bd	2.38	bd	9.12	4.9	1.5	32.6	19.4
Ag-Cp	1.35	0.35	0.65	0.53	bd	bd	bd	bd	bd	bd	15.12	bd	bd	6.56	17.2	46.2	82.9	58.9
Cs-Cp	1.32	0.34	0.66	0.63	bd	bd	bd	bd	bd	bd	bd	bd	20.97	4.26	19.0	47.7	82.6	51.2
NH_4_-Cp	1.23	0.43	0.26	0.42	bd	bd	bd	bd	bd	bd	bd	bd	bd	11.20	24.5	33.8	93.2	67.4

**Table 2:** The adsorption capacity of clinoptilolite for CH_4_ and N_2_ (at a pressure of approximately 1.01 × 10^5^ Pa at 298 K).

Adsorbent	qCH_4_/(mL/g)	qN_2_/(mL/g)	qCH_4_/qN_2_	qN_2_/qCH_4_
K-Cp	19.58	10.73	1.82	-
NH_4_-Cp	15.83	6.17	2.56	-
Cu-Cp	15.02	7.67	1.95	-
Rb-Cp	14.63	8.10	1.80	-
Zn-Cp	13.01	8.42	1.54	-
Ba-Cp	12.01	10.57	1.13	-
Cs-Cp	8.13	3.51	2.31	-
Li-Cp	4.97	10.17	-	**2.05**
Na-Cp	1.14	8.27	-	7.25
Ce-Cp	3.33	5.88	-	1.77
Sr-Cp	3.43	4.98	-	1.45
Ag-Cp	0.68	4.44	-	6.53
Mg-Cp	2.71	3.47	-	1.28
Ca-Cp	1.65	2.74	-	1.66
